# Closed Pantalar Dislocation With Checkrein Deformity: A Unique Case Report and Literature Review

**DOI:** 10.5435/JAAOSGlobal-D-20-00253

**Published:** 2021-10-21

**Authors:** Carl G. Speer, Richard H. Pike

**Affiliations:** From the Southeast Permanente Foot and Ankle Trauma and Reconstructive Surgical Fellowship, Atlanta, GA (Speer and Dr. Pike); and the Emerald Coast Foot and Ankle Center, Pensacola, FL (Speer).

## Abstract

Closed pantalar dislocations are a rare variant of an uncommon injury. Pantalar dislocations are typically caused by high-energy trauma resulting in an open injury with associated fracture of the articulating bones. Given its obscurity, the literature on closed pantalar dislocations is scarce, and no standard treatment protocol has been accepted. This case report chronicles the treatment and outcome of a 29-year-old man who presented with a checkrein deformity of all digits after a closed pantalar dislocation with 6-month follow-up. A comprehensive literature review found 28 articles representing 39 patients with closed pantalar dislocations without talar neck or body fractures. Roughly equal numbers of closed and open reduction techniques were performed with avascular necrosis occurring in 7 of 36 patients. Although outcome measures and follow-up were variable, what can be considered a suitable outcome was seen in approximately 83% of patients, with only 3 of 35 requiring a secondary operation. Long-term studies with well-defined outcome measures are needed to adequately predict the prognosis of this rare injury and efficacy of treatment protocols.

Defined as a pantalar dislocation, total talus dislocation, or enucleated talus, simultaneous dislocation of the talus about all three of its articulations is an extremely rare injury. Pantalar dislocations have been reported to occur in an estimated 2% to 10% of traumatic injuries of the talus^1,2^ and account for 0.06% of all dislocations.^3^ A pantalar dislocation without an open wound is a rare variant, reported to occur in only 15%^4^ of cases. This injury must be distinguished from total talar extrusions and talar neck fracture-dislocations. Although minor fracturing may occur, pantalar dislocations inherently lack the complications seen with talar reimplantation and internal fixation.

Because of its rarity, closed pantalar dislocations are often grouped with open injuries; however, these injuries are unique. Because less soft tissue is damaged with a closed injury, a lower incidence of talar avascular necrosis (AVN) is to be expected. Previous authors have suggested early total or partial talectomy^5^; however, subsequent research has shown that such aggressive measures are unneeded.^1-3,6,7,8,9,10,11,12,13,14,15,16,17,18,19,20,21,22,23,24,25,26,27^ The present study chronicles the authors' experience treating a checkrein deformity that resulted from a closed pantalar dislocation. To our knowledge, this study is the first to report such a deformity occurring after this injury. A literature review is included.

## Case Report

**Figure 1:**
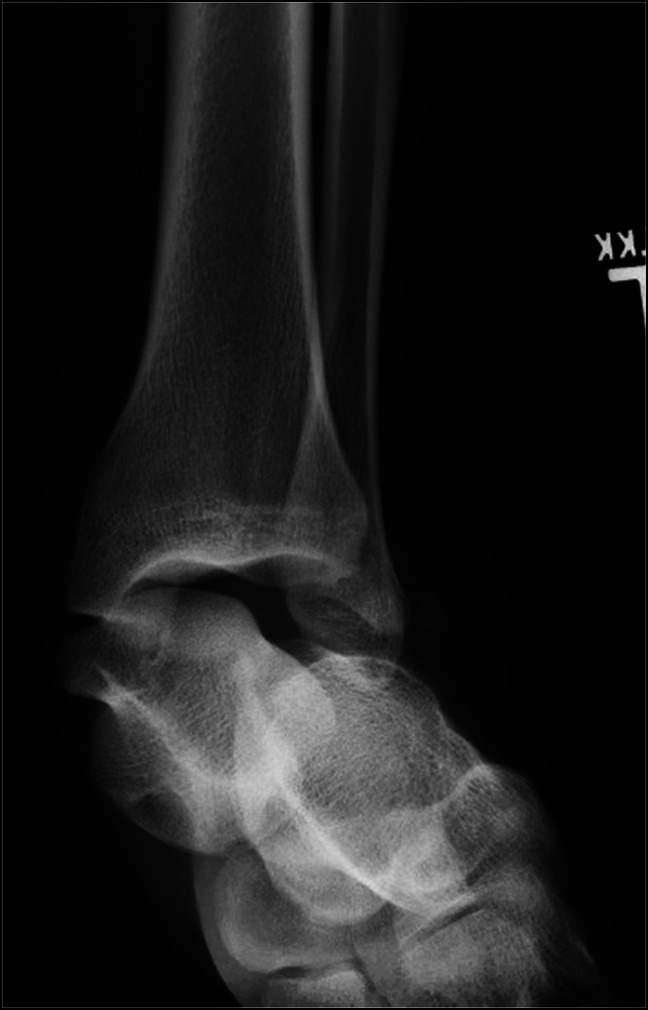
Prereduction anteroposterior ankle radiograph showing medial dislocation of the talus.

**Figure 2:**
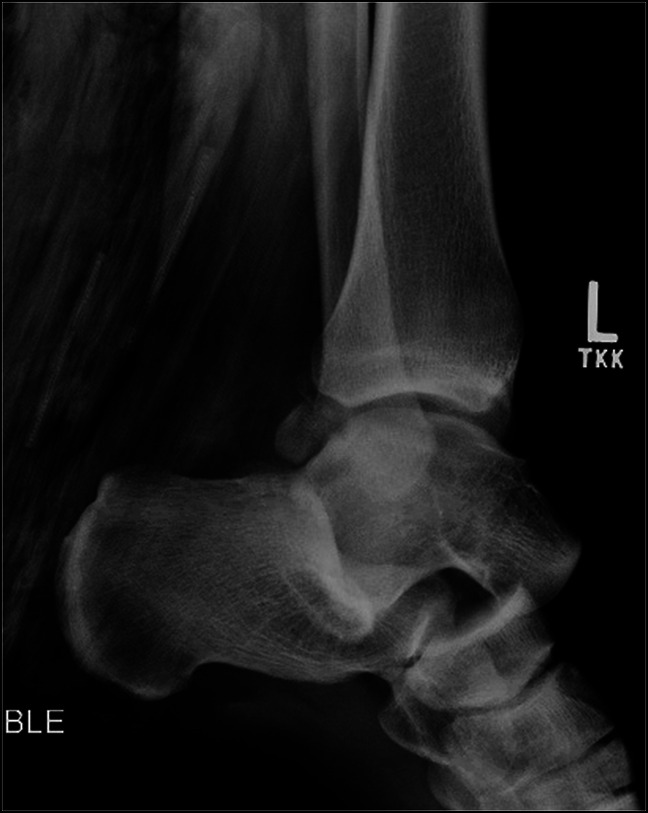
Prereduction lateral ankle radiograph showing anterior dislocation of the talus.

**Figure 3:**
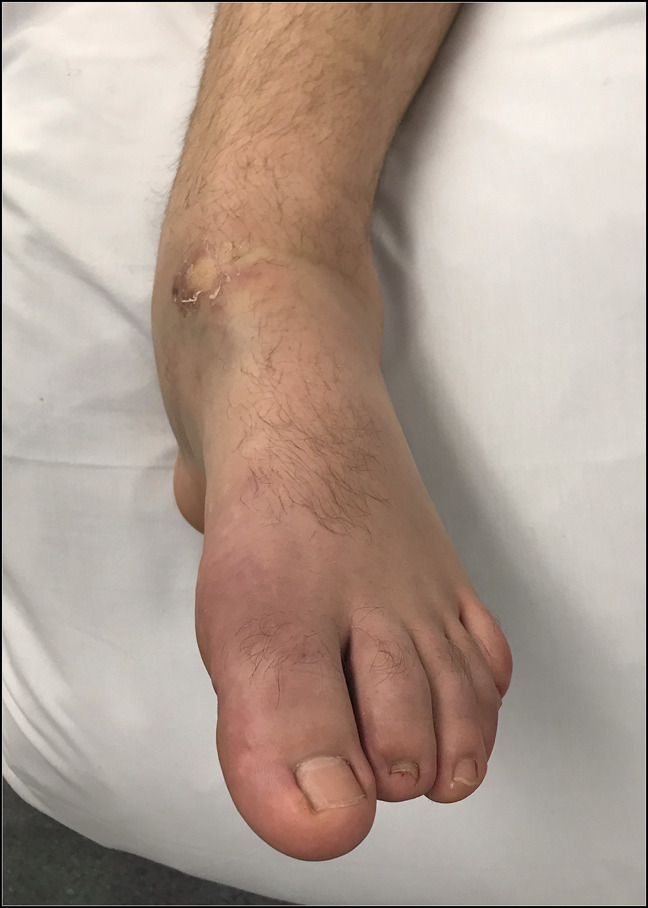
Prereduction photograph showing gross deformity of the ankle and rearfoot with plantar flexion checkrein deformity of the digits.

In January 2020, a healthy 29-year-old man presented to the emergency department with severe pain of the left lower extremity after falling approximately 20 feet from a ladder. The patient had no significant medical, family, social, or surgical history. He was on no medications and had no known drug allergies. Radiographs revealed anteromedial dislocation of the talus with minor fragmentation of the lateral malleolus and fracture of the posterolateral talar process (Figures 1 and 2). Closed reduction under conscious sedation was attempted without success by the emergency physician, and the foot and ankle team was consulted. On evaluation in the emergency department, the left foot and ankle appeared grossly swollen and deformed. No wounds, lacerations, or evidence of skin necrosis were seen. However, the skin was tented on the dorsal medial foot overlying the talar head. Pedal pulses were not palpable, but capillary refill was brisk in the digits and the foot was warm to touch. Sensation in the foot was intact to light touch. All five digits were plantarflexed at the interphalangeal joints, representing a checkrein deformity of the toes (Figure 3). The patient was able to actively flex and extend the digits. Compartments were soft and compressible. No other musculoskeletal injuries were identified. The patient was subsequently taken to the operating room for closed, possible open, reduction under anesthesia.

**Figure 4:**
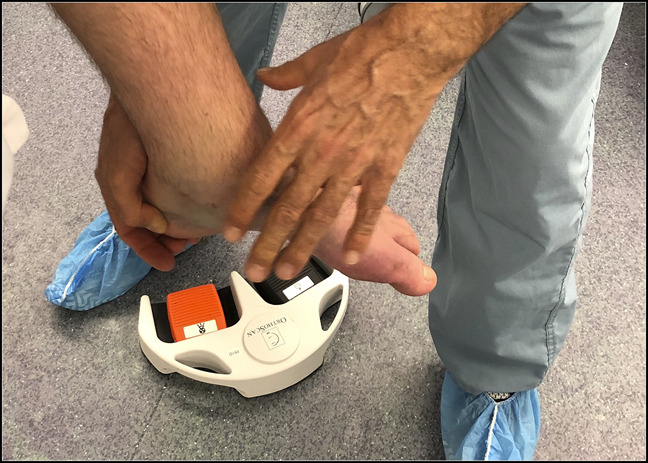
Photograph showing the hand position of the pushup type maneuver used for reduction.

**Figure 5:**
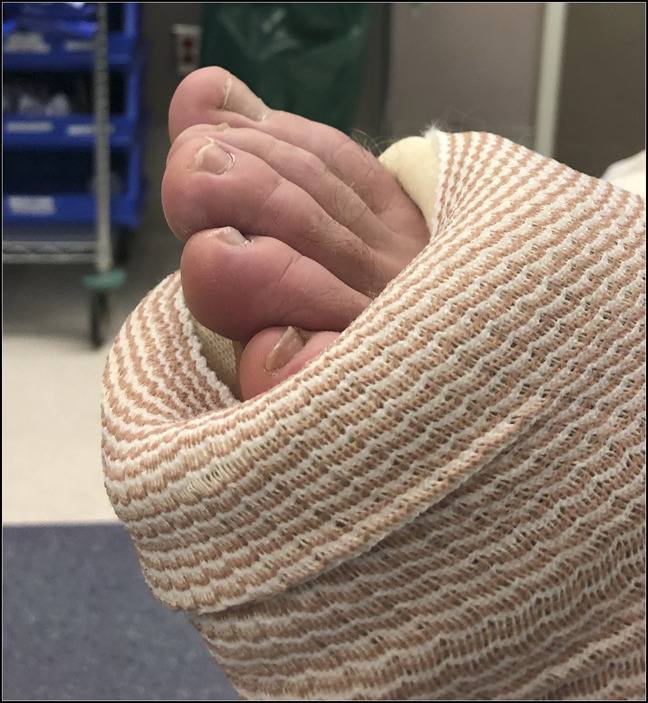
Postreduction and splinting of pantalar dislocation with resolution of digital checkrein deformity.

**Figure 6:**
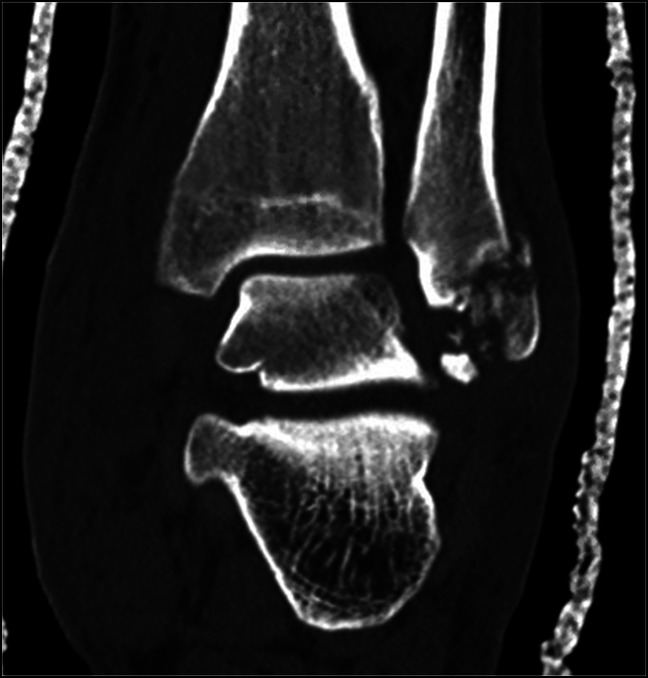
Immediate postreduction coronal CT image showing realignment of the ankle and subtalar joints with fracturing of the posterolateral fibula.

**Figure 7:**
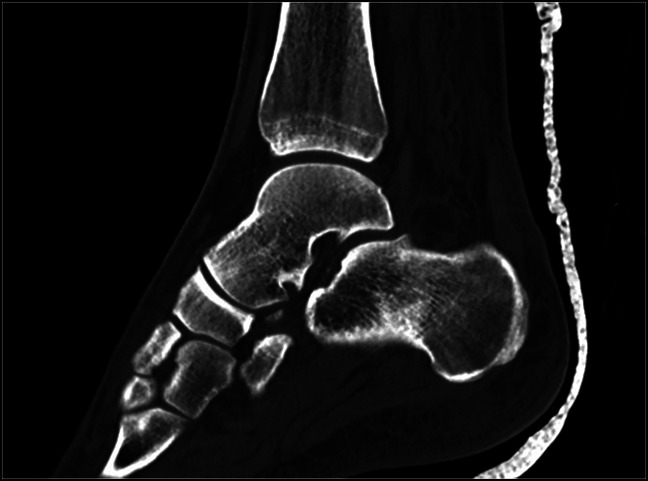
Immediate postreduction sagittal CT image showing realignment of the ankle, subtalar, and talonavicular joints.

**Figure 8:**
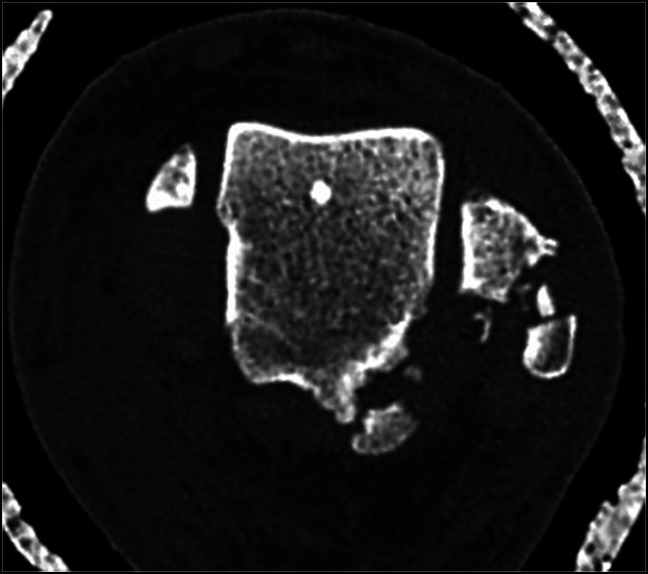
Immediate postreduction axial CT image showing comminuted fracture of the posterolateral talar process and posterolateral fibula.

After general anesthesia and relaxation was confirmed, the patient was positioned on the table in a supine position with the end of the table at the level of the knee joint so that the left leg was vertical and at 90° of knee flexion. The foot was grasped with one hand placed dorsally over the midfoot, talar head, and neck and the other hand encircling the medial, posterior, and lateral sides of the calcaneus. With this bimanual grasp, a pushup type maneuver was done pushing the foot toward the floor (Figure 4). With a modest amount of distraction placed on the foot, the subtalar and ankle joints reduced. Fluoroscopy confirmed reduction of those joints, but the navicular bone remained locked beneath the plantar lateral edge of the talar head. With this in mind, the forefoot was first slightly plantarflexed and then distracted and pulled dorsally and turned into supination. With this technique, the talonavicular joint was reduced. Then, while holding the hallux in dorsiflexion and foot in supination, fluoroscopy confirmed reduction of the talonavicular joint and the subtalar, and tibiotalar joints. After complete reduction, all toes were relaxed in a neutral position, the checkrein mechanism having been eliminated. Xeroform gauze was placed over the area of skin tenting, followed by a stockinette, bulky soft-tissue dressing, and posterior and stirrup plaster secured with an ACE wrap (Figure 5). The patient was admitted postoperatively for 2 days for pain management, neurovascular checks, and physical therapy consultation. Postreduction CT imaging obtained during the hospitalization revealed maintenance of joint reduction with comminuted fracture to the posterolateral fibula and posterolateral process of the talus (Figures 6–8).

The patient remained non–weight bearing in the splint for 2 weeks. At 2 weeks, the splint was removed, and non–weight bearing was continued in a tall pneumatic medical boot. The patient was allowed active range of motion exercises out of the boot at 3 weeks. At 6 weeks postreduction, he was allowed minimum weight bearing in the boot around the house only. The patient was walking without the boot at 10 weeks with some pain. A follow-up CT scan, 12 weeks after reduction, again revealed mild comminution to the posterolateral distal fibula with surrounding small bone fragments but no intra-articular loose bodies.

**Figure 9:**
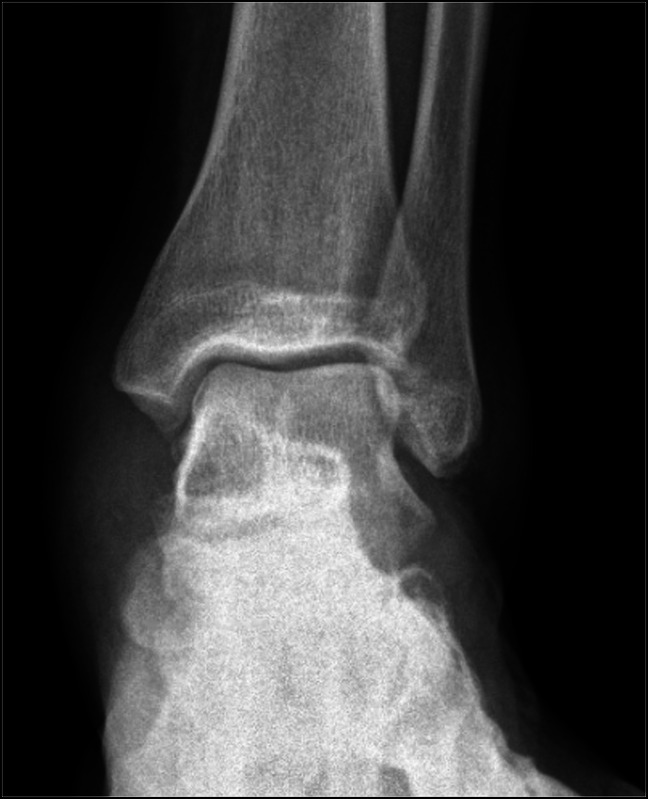
Anteroposterior weight-bearing radiograph image 6 months postreduction without signs of avascular necrosis of the talus.

**Figure 10:**
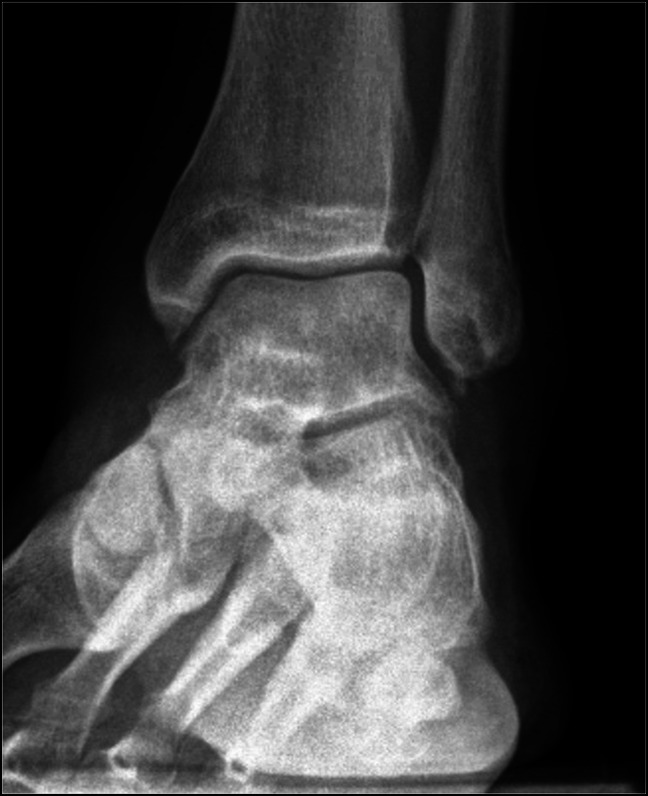
Ankle mortise weight-bearing radiograph image 6 months postreduction without signs of avascular necrosis of the talus.

**Figure 11:**
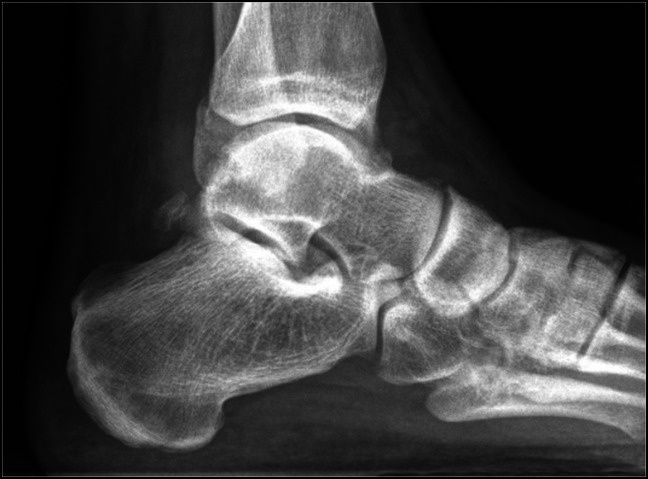
Lateral weight-bearing radiograph image 6 months postreduction showing maintained joint alignment and posterior fibula fracture union.

He was last evaluated 6 months after his injury. At that time, he related having mild pain and swelling with limitation in recreational activities. He was ambulating without assistive devices. No tenderness was provoked on palpation of the posterior talus or the distal fibula. Radiographic union of the posterolateral distal fibula fracture was seen along with no evidence of AVN of the talus (Figures 9–11). At this last follow-up, he had approximately 20° of motion of both the ankle and subtalar joints. His AOFAS hindfoot score was 81.

## Discussion

A closed, pantalar dislocation without associated talar neck or body fracture is a rare variant of an uncommon injury. Although the talus has no muscular or tendinous attachments, the congruency of its joint surfaces and surrounding ligaments provides adequate stability. Thus, to dislocate the talus from all three of its articulations, a significant force is required, which typically results in an open injury and/or fracture of the talus. Scarce literature describing a closed pantalar dislocation without an accompanying major talus fracture exists. The current case report is unique in that the injury was associated with a checkrein deformity of the digits.

As with all dislocations, urgent reduction and maintenance of anatomic alignment is the key goal of treatment. In this case, closed reduction via manual traction and plaster splinting resulted in realignment and stability. However, reducing a pantalar dislocation is often not this simple. When manual traction does not suffice, an axially placed calcaneal Steinmann pin or Kirschner wire may be needed for longitudinal traction.^6,16,20,28,29^ If redislocation after reduction is suspected, Kirschner wire and/or Steinmann pin transfixation across the joints can be used.^3,10,12,18,20,22,26,28,29^ External fixation may be used as well.

When closed reduction is unsuccessful, soft-tissue and/or bone impingement is likely preventing the reduction. In the case of closed, pantalar dislocations, joint capsule,^13^ long flexor tendons,^13,26^ and bone fragments^3^ have been described as preventing closed reduction. In such cases, open reduction becomes necessary. Incision placement should be planned to maximize exposure and avoid neurovascular structures. Approaches via an anteromedial,^9,24^ anterolateral,^17^ combined anteromedial and lateral,^3,13,14,26^ and dorsal^15^ incision have been reported.

This report is the first to document a simultaneous checkrein deformity of the great and lesser toes after a pantalar dislocation. Checkrein deformity of the great toe secondary to entrapment of the flexor hallucis longus tendon has been described after fractures of the talus and ankle and after fibular graft removal.^30-32^ Similar to our case, Tucker et al^33^ noticed slight plantar flexion of the toes at the metatarsal-phalangeal and interphalangeal joints after a closed, lateral subtalar joint dislocation. However, a checkrein deformity was not diagnosed in this case. Tanwar et al^34^ diagnosed a checkrein deformity of the great toe after a closed lateral subtalar joint dislocation. They concluded that the great toe was hyperflexed because the flexor hallucis longus tendon was tethered against the inferomedially dislocated talar head. It can be assumed that with a checkrein deformity of all the toes, both the flexor hallucis longus and flexor digitorum longus tendons become trapped under the dislocated talar head.

As noted in the table, a closed, pantalar dislocation has no standard postoperative protocol. However, treatment can be guided from the literature on closed subtalar and ankle joint dislocations. Good results have been reported with 4 weeks of non–weight-bearing immobilization, followed by progressive range of motion and weight bearing after subtalar joint dislocations.^35-37^ After a closed ankle joint dislocation, non–weight bearing is often maintained for 6 to 8 weeks with progression as tolerated.^38-40^ In our case, non–weight bearing for 6 weeks with range of motion at 3 weeks allowed for soft-tissue healing while simultaneously limiting joint stiffness. Rehabilitation and weight bearing progression should be guided by clinical and radiographic evidence of healing. If pain continues, CT may be needed to assess joint alignment, fracture healing, and loose intra-articular bone fragments.^27^ MRI can help evaluate soft tissue possibly damaged in this injury such as joint capsule, deltoid ligament, calcaneofibular ligament, and anterior talofibular ligament. In all cases, a postreduction CT is recommended so as to not miss any associated fractures.

Previously reported range of motion data are difficult to interpret because most studies do not use the contralateral ankle or subtalar joint as a control and preinjury data are not available. When looking at range of motion after closed ankle and subtalar joint dislocations in isolation, range of motion of the joints has been shown to decrease,^36,38,41^ causing various levels of disability.^36-38,42,43^ Ankle joint dorsiflexion after a closed pantalar dislocation has been reported to range from −5° to 20°, whereas plantar flexion ranges from 20° to 40°.^2,16,26,28,29^ At 6 months, the current patient had 8° of dorsiflexion and 10° of plantar flexion on the injured extremity. The uninjured ankle joint had 13° of dorsiflexion and 30° of plantar flexion. Yapici et al^3^ mention a 5° loss in subtalar joint inversion and eversion, whereas Newcomb and Brav^29^ measured 10° of inversion and 5° eversion after this injury. At last follow-up, the current patient had 15° of inversion of the treated extremity, whereas the contralateral side had 10°. No appreciable difference in subtalar joint eversion was found between the two sides.

To our knowledge, only two studies have reported AOFAS scores after a closed pantalar dislocation. Itsiopoulos et al^15^ reported a score of 88 at 6 months, whereas Gopisankar Balaji et al^12^ found a score of 78 at 1 year despite an associated Lisfranc injury. At 6 months after closed reduction, the current patient had an AOFAS hindfoot score of 81. As a comparison, AOFAS scores after a closed, isolated subtalar joint dislocation range from 80 to 97.^33,35,36,41,43^ It is surprising that AOFAS scores after closed pantalar dislocation are similar to those after closed subtalar joint dislocation despite an additional joint being affected in the former. However, further studies are needed to assess the functional outcomes of closed pantalar dislocations.

The long-term complications after a closed pantalar dislocation are uncertain. As is the current case, most literature has been limited to case studies with short follow-up and subjective outcome measures. Furthermore, open and closed injuries often get grouped together without differentiating results between the two.^4,44^ Some authors^8,21,25,27^ have reported on clinical results per criteria defined by Kenwright and Taylor^45^ with mixed results. Previously reported complications include joint stiffness,^12,14,20^ pain,^8,16,21^ AVN of the talus,^16,21^ deep vein thrombosis,^14^ and Sudeck atrophy.^6^

The occurrence of AVN is associated with the loss of soft-tissue attachments and disruption of the intraosseous blood supply. Because approximately 60% of the talus is covered by cartilage, there is limited surface area for nutrient vessels to penetrate. Talar blood supply comes from branches of the peroneal, dorsalis pedis, and posterior tibial arteries. The artery of the tarsal canal, arising medially from the posterior tibial artery, anastomosis inferior to the talus with the artery of the tarsal sinus, which arises laterally from the anterior tibial and perforating peroneal vessels. This anastomotic sling enters the talar body inferiorly. The posterior tibial artery also provides a deltoid branch, which supplies the medial talar body.^46^ The posterior process is supplied via small vessels from the posterior tibial and peroneal arteries.^46,47^ The neck is supplied superiorly from branches of the anterior tibial artery.^46^ Newcomb and Brav^29^ stated that the major nutrient supply is through the superior talonavicular ligament and when strands of the ligament remain attached revascularization occurs. On the contrary, Sharifi et al^24^ argued that because all capsular and ligamentous attachments are severed with this injury, blood supply is likely retained through the intraosseous deltoid branch or posterior process branch to the talus. Others have concluded that poor prognosis is inevitable when the inferior blood supply through the anastomotic sling is disrupted.^22^

Given its tenuous blood supply, AVN of the talus after a pantalar dislocation is concerning. In their systematic review, Weston et al^4^ found a 24% incidence of AVN among patients without associated fracture of the talus, foot, or ankle. However, in this study, no subgroup analysis between open and closed injuries was done. Newcomb and Brav^29^ concluded that revascularization occurs more readily if the talar neck is not fractured, whereas some authors^4,5^ have proposed that AVN is likely even in the absence of significant fracture. However, even when AVN has been diagnosed, results are not always poor. Boden et al^9^ reported on 19 pantalar dislocations without a fracture of the talus, of which 5 were closed injuries. Among the closed injuries, there was an average of 22 months of follow-up. Postoperative radiographs revealed four instances of osteonecrosis of the talus and two cases of subtalar joint arthrosis. Despite these findings, no cases of collapse of the talus or secondary procedures were documented. Furthermore, the incidence of osteonecrosis and posttraumatic arthrosis was not related to having an open or closed injury. Good results were also seen despite AVN by Katz and Yang.^16^ In their article, the patient did have some pain at 2 years after the injury but was ambulating without assistance and did not need further surgery. Ritsema^21^ reported on five female patients with a closed pantalar dislocation with an average 4.5-year follow-up. Two of the patients developed AVN. One case was treated by ankle joint arthrodesis at 2 months with fair results, whereas the other had good results by staying non–weight bearing until the necrotic bone remodeled.

The Hawkins sign, the subchondral radiolucent band seen beneath the talar dome, is often used as a prognostic indicator of revascularization of the talus. After a talus fracture, the Hawkins sign usually appears 6 to 9 weeks after injury and has a 57.7% specificity and a 100% sensitivity^48^ for talus vascularization. The specificity and sensitivity, and at what time they appear, are not determined in the absence of fracture of the talus. Although not seen in the current case report, the Hawkins sign has been identified after a pantalar dislocation.^6,22,26^ While a good indicator of vascularization, the lack of a Hawkins sign does not predict AVN.

The current case report details our experience treating a closed pantalar dislocation that was accompanied by a unique checkrein deformity. Fortunately, the dislocation and checkrein of the digits resolved with closed reduction and plaster splinting. When closed reduction fails, open reduction is urgent,^49^ and transfixion wires or pins can be used if concerned for redislocation. Although there is no standard postoperative protocol, our patient did well with 6 weeks of non–weight bearing and active range of motion at 3 weeks. Six months after the injury, the patient was walking unassisted without clinical or radiographic signs of AVN, but with some restrictions in athletic activity. Because the patient was asymptomatic, an MRI was not done; however, this may have provided a more thorough analysis. Furthermore, any conclusions on the results of our patient are guarded as AVN risk can remain for up to 3 years.^22^ Long-term studies with a larger sample size and objective outcome measures are needed to better understand the prognosis of this rare injury. We intend to reevaluate at 1-, 2-, and 3-year follow-up intervals.

## Literature Review

A comprehensive literature review was conducted using the PubMed, MEDLINE, and Embase databases to search the following: “Talar dislocation OR talus dislocation OR total talar dislocation OR total talus dislocation OR talus enucleation OR talar enucleation OR pantalar dislocation.” Articles with human subjects that were published in the English language were included. Articles were then further screened to include only those detailing closed pantalar dislocations without fracture of the talar neck or body. These exclusions were made because patients with open talar dislocations and/or a major talus fracture require different treatment from the closed variety with absence of fracture.

Thirty articles met our inclusion criteria; however, only 28 were retrievable, representing 39 patients (Table 1). There were 28 men and 11 women, with an average age of 34 years (range: 19 to 68 years). The average follow-up time was 28 months; ranging from 6 months to 9 years. The etiology of pantalar dislocation was reported in 34 patients. Motor vehicle accidents caused most injuries (53%), with fall from a height contributing 35% and 12% resulting from other high-impact injuries. Twenty-nine patients had the direction of dislocation reported, with 15 (52%) dislocated anterolateral, 8 lateral (27.5%), 3 medial (10%), 2 dorsal (7%), and 1 posterior (3%). The reduction technique was recorded in all 39 patients, with 21 reduced in a closed fashion and 18 requiring open reduction. As noted in the table, ancillary Steinmann pins for longitudinal traction and transfixion Kirschner wires for postreduction stabilization were often used. The occurrence or nonoccurrence of AVN of the talus was reported in 36/39 patients. Overall, 7/36 patients developed AVN postoperatively. In four of these seven cases, the term osteonecrosis as defined by Boden et al^9^ was used as a surrogate for avascular necrosis. Equating documented findings of osteonecrosis with AVN helps explain our higher incidence of this sequela compared with that reported by Vosoughi and Vallier.^49^ The final outcome was reported in 35/39 patients. Given the heterogeneous nature of the included studies, a broad definition of a suitable outcome was needed. Outcomes described by the authors as being asymptomatic, normal, pain-free, no arthrosis, good, fair, and/or no AVN were categorized as a suitable outcome. Using these parameters, a suitable outcome was achieved in 83% of patients. Three patients had continued arthrosis, sclerosis, and/or pain, whereas an additional three patients required a secondary operation at final follow-up.

**Table 1:** Published Reports of Closed Pantalar Dislocation With Associated Patient Information (N = 28 Articles, Representing 39 Patients)

Author/Year	Cases	Sex	Age (yr)	Etiology	Direction	Reduction	Associated Fractures	Postoperative Protocol	AVN	Hawkins Sign/Time Postoperative	Outcome
Alaoui and Kassou,^10^ 2016	1	M	32	MVA	Anterolateral	Closed with Kirschner wire stabilization	Lateral malleolus	Cast boot for 2 mo, followed by PT	No	No mention	Ankle painless and stable with satisfactory mobility at 18 mo
Alrashidi et al,^6^ 2014	1	M	30	Fall from height	Anterolateral	—	4th metatarsal base	Back slab, ROM then mobilized in a wheelchair as pain/swelling subsided, and PT with FWB at 8 wk	No	Yes, 6 mo	Sudeck atrophy at 3 mo and asymptomatic at 1 yr
Aranganathan and Dharmarajan,^7^ 2011	1	F	25	Fall from height	No mention	Closed	Lateral malleolus	No mention	No	No mention	No AVN at 1 yr
Balaji et al,^12^ 2012	1	M	35	Fall from height	No mention	Closed with Kirschner wire stabilization	Lisfranc injury and fracture 3–5 metatarsal bases	NWB splint 10 d, NWB cast for 3 mo, and ankle ROM with gradual ambulation with crutches	No	No mention	AOFAS: 78, 30° plantar flexion and terminal restriction of dorsiflexion, and painless ankle ROM at 1 yr
Bas et al,^8^ 1994	2	M	No mention	Fall from height	Medial	Closed	No	No mention	No	No mention	Required triple arthrodesis; fair at 4 yr (clinical results per Kenwright and Taylor^45^ criteria)
—	—	M	No mention	Fall from height	Lateral	Closed	Medial malleolus fracture	No mention	No	No mention	Required blair arthrodesis at 2 mo, poor at 3 yr (clinical results per Kenwright and Taylor^45^ criteria)
Boden et al,^9^ 2017	5	F	68	MVA	No mention	Open (unable to determine whether Kirschner wire stabilization and/or external fixation was used)	No	NWB for 12 wk advised	Yes	No mention	STJ arthrosis at 11 mo
—	—	M	19	Fall from height	No mention	Closed (unable to determine whether Kirschner wire stabilization and/or external fixation was used)	Bimalleolar ankle fracture	NWB for 12 wk advised	Yes	No mention	No arthrosis at 48 mo
—	—	F	33	MVA	No mention	Closed (unable to determine whether Kirschner wire stabilization and/or external fixation was used)	Calcaneal and tibial shaft fracture	NWB for 12 wk advised	Yes	No mention	STJ arthrosis, MFA: 37, FFI: 20 at 25 mo
—	—	M	28	MVA	No mention	Open (unable to determine whether Kirschner wire stabilization and/or external fixation was used)	No	NWB for 12 wk advised	No mention	No mention	No mention
—	—	M	25	MVA	No mention	Open (unable to determine whether Kirschner wire stabilization and/or external fixation was used)	Femoral shaft and medial malleolus fracture	NWB for 12 wk advised	Yes	No mention	No arthrosis, MFA: 27, FFI: 23 at 24 mo
Condes et al,^22^ 2003	1	M	25	MVA	Anteromedial	Open with Kirschner wire stabilization	Lateral malleolus marginal fracture	NWB cast for 2 mo and WB cast for 1 mo	No	Yes, 2 mo	Normal ROM and function at 3 yr
Dulani et al,^28^ 2012	1	M	19	Fall from height	Anterolateral	Open with a calcaneal Steinmann pin for traction and Kirschner wire stabilization	Medial malleolus and posterior talar tubercle	NWB cast for 6 wk and NWB with gradual mobilization for another 6 wk	No	No mention	Pain-free ankle joint ROM with 10° dorsiflexion and 40° plantar flexion at 1 yr
Gaskin and Pimple,^11^ 2007	2	M	45	Twisted foot while go-carting	No mention	Closed	No	NWB 6 wk, PWB in the air cast boot with PT for 6 wk, and FWB	No	No mention	Asymptomatic with normal ROM at 1 yr; no AVN or arthritis on radiograph at 2 yr
—	—	M	43	Fell from motorbike	No mention	Closed	No	NWB 6 wk, PWB in the air cast boot with PT for 6 wk, and FWB	No	No mention	Asymptomatic with normal ROM at 1 year; no AVN or arthritis on radiograph at 2 yr
Gursu et al,^13^ 2013	1	M	25	Fall from height	Anterolateral	Open	No	NWB 6 wk and then PT and FWB	No	No mention	No pain, ankle with the same ROM as unaffected side at 2 yr
Hendin and Rosenberg,^50^ 2016	1	M	36	MVA	Dorsal	Closed	Posterior talar process	No mention	No mention	No mention	No mention
Heylen et al,^14^ 2011	1	M	46	Fall from height	Anterolateral	Open	Small fractures to lateral and medial malleolus	NWB in the below-knee cast for 6 wk and PWB in the ROM walker increasing to FWB with PT at 9 wk	No	No mention	DVT at 9 wk and normal plantar flexion with slightly decreased dorsiflexion at 1 yr
El Ibrahimi et al,^1^ 2011	1	F	34	Fell off motorbike	Anterolateral	Closed	Lateral malleolus	Plaster for 2 mo and PT	No	No mention	Painless and stable ankle with satisfactory mobility
Itsiopoulos et al,^15^ 2018	1	M	25	Fell off motorbike	Anterolateral	Open	No	NWB for 8 wk in the below-knee splint, PWB with crutches at 9 wk, and PT at 11 wk	No	No mention	Asymptomatic with good ROM and AOFAS: 88 at 6 mo
Katz and Yang,^16^ 2000	1	M	34	MVA	Posterior	Closed with a Steinmann pin for traction	No	NWB in the cast for 16 wk and ROM exercises with minimal weight bearing	Yes	None	Ambulated without assistance, dorsiflexion: −5° and plantar flexion: 20° with some pain at 2 yr
Korovessis et al,^17^ 1992	1	M	33	MVA	Lateral	Open with Steinmann pin stabilization	No	NWB in the splint for 6 wk and weight bearing at 8 wk	No	No mention	Asymptomatic with no ROM limitation at 3 mo and no arthritic changes on radiograph at 2 yr
Kumar et al,^18^ 2014	1	M	25	MVA	Anterolateral	Closed with Kirschner wire stabilization	No	Below-knee splint for 6 wk and mobilized	No	No mention	Good ROM at 1 yr
Nanjayan et al,^19^ 2014	1	M	19	Fell from bike	Anterolateral	Closed	5th metatarsal base	NWB for 6 wk and FWB with PT	No	No mention	Full, pain-free ROM with no features of arthritis at 2 yr
Newcomb and Brav,^29^ 1948	1	M	45	Jumped from moving vehicle	Anterolateral	Closed using Kirschner wire and Steinmann pin for traction	No	NWB for 4 wk in the toe-to-groin plaster and active exercise stared	No	No mention	No aseptic necrosis, 30° plantar flexion, 10° dorsiflexion, 5° eversion, and 10° inversion at 6 mo
Papanikolaou et al,^20^ 2002	1	M	28	MVA	Anterolateral	Closed with Steinmann pin stabilization	No	After 6 wk, the Steinmann pin removed and ROM started, at 3 mo allowed gradually to bear weight	No	No mention	Pain-free and unrestricted motion at 2.5 yr except for last 5° of supination/pronation
Rhanim et al,^2^ 2014	1	F	29	Fell off motorbike	Lateral	Closed	No	NWB for 5 wk, PWB in the air cast boot and PT at 6 wk, and FWB without the boot at 3 mo	No	No mention	Pain free with satisfactory motion (15° dorsiflexion/30° plantar flexion) at 1 yr
Ritsema,^21^ 1988	5	F	50	No mention	Medial	Open	No	No mention	Yes	No mention	Required tibiotalar joint fusion, fair at 3 yr (clinical results per Kenwright and Taylor^45^ criteria)
—	—	F	24	No mention	Lateral	Open	No	No mention	No	No mention	Good at 3 yr
—	—	F	20	No mention	Lateral	Open, required tenotomy of the peroneal tendon	No	No mention	Yes	No mention	Good at 6 yr
—	—	F	60	No mention	Lateral	Open	No	No mention	No	No mention	Good at 4 yr
—	—	F	39	No mention	Medial	Open, required tenotomy of the anterior tibial tendon	No	No mention	No	No mention	Fair at 4 yr
Segal and Wasilewski,^23^ 1980	1	M	50	MVA	Anterior	Closed	No	NWB cast for 6 wk	No	No mention	Asymptomatic at 13 mo
Sharifi et al,^24^ 2009	1	M	28	Fall from height	Lateral	Open	Cuboid	PWB with toe touch at beginning and FWB at 3 mo with PT	No	None seen	No AVN or posttraumatic arthritis at 1 yr
Taymaz and Gunal,^25^ 2005	1	M	49	Fall from height	No mention	Closed	No	NWB in the cast for 6 wk and FWB	No	No mention	Excellent at 9 yr (clinical results per Kenwright and Taylor^45^)
Turhan et al,^26^ 2012	1	M	25	Ankle sprain	Lateral	Open with Kirschner wire stabilization and tibialis posterior tendon entrapment in the ankle joint	Small fleck of the medial talus	NWB in the cast for 6 wk, mobilized with PWB using crutches and PT, and FWB at 10 wk	No	Yes, at 16 wk	20° dorsiflexion, 40° plantar flexion at 16 wk, and walking with no pain/instability at 1 yr
Xarchas et al,^27^ 2009	2	F	35	MVA	Anterolateral	Closed	No	NWB in the cast for 6 wk, PWB with the walker for 1 wk, 1-wk walking with 2 crutches, 1 wk with 1 crutch, and FWB at 9 wk	No	No mention	Excellent (clinical results per Kenwright and Taylor^45^) with >90% ROM in the AJ and STJ at 3 yr
—	—	M	45	MVA	Anterolateral	Closed	No	No mention	No mention	No mention	No mention (lost to follow-up)
Yapici et al,^3^ 2019	1	M	32	Fall from height	Anterolateral	Open with Kirschner wire stabilization	Medial malleolus	NWB for 6 wk in the cast and walk with crutches at 6 wk	No	None seen	Pain-free walking at 3 mo, 10° flexion and extension loss, and 5° inversion and eversion loss at 36 mo

AJ = ankle joint; AOFAS = American Orthopaedic Foot and Ankle Society; AVN = avascular necrosis; DVT = deep vein thrombosis; FFI = Foot Function Index; FWB = full weight bearing; MFA = musculoskeletal function assessment; MVA = motor vehicle accident; NWB = non–weight bearing; PT = physical therapy; PWB = partial weight bearing; ROM = range of motion; SJ = subtalar joint; WB = weight bearing
